# A cluster randomized trial to improve adherence to evidence-based guidelines on diabetes and reduce clinical inertia in primary care physicians in Belgium: study protocol [NTR 1369]

**DOI:** 10.1186/1748-5908-3-42

**Published:** 2008-10-06

**Authors:** Liesbeth Borgermans, Geert Goderis, Carine Van Den Broeke, Chantal Mathieu, Bert Aertgeerts, Geert Verbeke, An Carbonez, Anna Ivanova, Richard Grol, Jan Heyrman

**Affiliations:** 1 Catholic University Leuven, Department of General Practice, Kapucijnenvoer 33/J Box 7001, 3000 Leuven, Belgium; 2 University Hospitals Leuven, Experimental Medicine, Herestraat 49, 3000 Leuven, Belgium; 3 Catholic University Leuven, Leuven Statistics Research Centre (LStat), Celestijnenlaan 200 B, 3001 Heverlee, Belgium; 4 Radboud University of Nijmegen, Faculty of Medicine, Centre for Quality of Care, PO BOX 9101, KWAZO 114, 6500 HB Nijmegen, The Netherlands

## Abstract

**Background:**

Most quality improvement programs in diabetes care incorporate aspects of clinician education, performance feedback, patient education, care management, and diabetes care teams to support primary care physicians. Few studies have applied all of these dimensions to address clinical inertia.

**Aim:**

To evaluate interventions to improve adherence to evidence-based guidelines for diabetes and reduce clinical inertia in primary care physicians.

**Design:**

Two-arm cluster randomized controlled trial.

**Participants:**

Primary care physicians in Belgium.

**Interventions:**

Primary care physicians will be randomly allocated to 'Usual' (UQIP) or 'Advanced' (AQIP) Quality Improvement Programs. Physicians in the UQIP will receive interventions addressing the main physician, patient, and office system factors that contribute to clinical inertia. Physicians in the AQIP will receive additional interventions that focus on sustainable behavior changes in patients and providers.

**Outcomes:**

Primary endpoints are the proportions of patients within targets for three clinical outcomes: 1) glycosylated hemoglobin < 7%; 2) systolic blood pressure differences ≤130 mmHg; and 3) low density lipoprotein/cholesterol < 100 mg/dl. Secondary endpoints are individual improvements in 12 validated parameters: glycosylated hemoglobin, low and high density lipoprotein/cholesterol, total cholesterol, systolic blood pressure, diastolic blood pressure, weight, physical exercise, healthy diet, smoking status, and statin and anti-platelet therapy.

**Primary and secondary analysis:**

Statistical analyses will be performed using an intent-to-treat approach with a multilevel model. Linear and generalized linear mixed models will be used to account for the clustered nature of the data, *i.e*., patients clustered withinimary care physicians, and repeated assessments clustered within patients. To compare patient characteristics at baseline and between the intervention arms, the generalized estimating equations (GEE) approach will be used, taking the clustered nature of the data within physicians into account. We will also use the GEE approach to test for differences in evolution of the primary and secondary endpoints for all patients, and for patients in the two interventions arms, accounting for within-patient clustering.

**Trial Registration:**

number: NTR 1369.

## Background

Diabetes management is a complex process requiring physiological, psychological, and social interventions [1,2]. Although considerable evidence supports the use of pharmacological interventions in diabetes care [3,4], the best way to improve health outcomes using non-pharmacological 'complex interventions' is often unclear [5]. A number of complex interventions target improvements in patient, provider, and organizational aspects of diabetes care [6]. The active components of these complex interventions are essential to their proper functioning and may act both independently and interdependently [7]. The Chronic Care Model (CCM) is often used as a conceptual framework to underpin complex interventions in diabetes care [8,9]. According to this model, patient outcomes such as good control of risk factors are associated with the presence of one or more interrelated components: community resources, self-management support, delivery system redesign, decision support, clinical information systems, and organizational support [8]. Most quality improvement programs in diabetes care cover several dimensions of the CCM, in particular those supported by substantial evidence of improved outcomes of care in selected populations [10]. Clinician education and dissemination of guidelines [11,12], feedback on performance [13], patient education [14,15], care management [16,17], and diabetes care teams (DCTs) to support primary care physicians [18-20] represent examples of such interventions. Few studies have applied all dimensions of the CCM to address non-adherence to evidence-based guidelines and to reduce 'clinical inertia' in primary care physicians [21-23].

Clinical inertia is defined as a lack of treatment initiation or intensification in a patient that is not achieving evidence-based goals of care [24]; this is consistent with the definition of medical errors given by the Institute of Medicine [25,26]. Clinical inertia increases the likelihood of adverse outcomes in a high proportion of patients, but it may take years for poorer clinical outcomes to become apparent [27]. Numerous authors, including those who report on clinical inertia, have defined three principal sources for non-adherence to evidence-based guidelines and clinical inertia: physician factors, patient factors, and office system and organizational factors [28-30]. Physician factors that contribute to clinical inertia include an overestimation of care actually delivered, a failure to identify and manage comorbid conditions, disagreement with evidence-based goals of care and the use of 'soft reasons' to avoid intensification of therapy (*e.g*., patient refusal) [31,32]. Patient factors that contribute to clinical inertia are limited motivation or resistance to adopting lifestyles that support optimal disease care, which stresses the importance of patient empowerment as a cornerstone to high-quality diabetes care [33,34]. Office system and organizational factors that contribute to clinical inertia are the absence of decision support and a team approach to care. These three sources interact in complex ways, and interventions to reduce clinical inertia therefore need to be multifactorial in nature. Here, we describe a study protocol of a cluster randomized trial. We have chosen the physician's practice as the unit of randomization since this was considered the most feasible method of conducting the trial. We plan to compare two different interventions for improving adherence to evidence based guidelines and reducing clinical inertia in primary care physicians.

### Aim of the study

Our program goal is to improve adherence to evidence-based guidelines and to reduce clinical inertia in primary care physicians, and to therefore improve the management of glycemic control and cardio-vascular risk factors in persons with diabetes.

### Scientific hypothesis

One hypothesis is that an advanced quality improvement program (AQIP) significantly improves clinical outcomes in persons with type 2 diabetes compared to a usual quality improvement program (UQIP). Subgroup analyses can analyze the effect of the program in the two intervention arms using cut-off values. The second hypothesis is that persons with type 2 diabetes who make use of a DCT will have significantly better outcomes compared to non-users of the DCT, regardless of their intervention arm.

## Methods

### Study design

The study is an open pragmatic cluster randomized trial with before/after measurements and two intervention arms. A cluster design is necessary because randomization is performed on a practice level, the intervention happens on the physician level, but a large part of the data are analyzed at the patient level. The implementation period of the trial is 18 months.

### Participants

All 379 active primary care physicians (PCPs) in the project region are invited to participate in the project. These PCPs work in a semi-rural setting with 357,000 inhabitants and serve predominantly Caucasian patients with type 2 diabetes mellitus. PCPs provide care for approximately 80% of patients with type 2 diabetes, and are often the sole providers of care. The only inclusion criterion for the providers is agreeing to recruit all patients with type 2 diabetes mellitus to prevent selection bias. In addition, PCPs will be asked to screen more systematically for new type 2 diabetes mellitus patients during the seven months after registration begins. Diabetes is defined in accordance with the 2003 ADA criteria [35] with PCPs making the final diagnosis.

Only patients with type 2 diabetes mellitus will be included in the study, regardless of age. Patients who cannot provide informed consent will be excluded from the study.

### Intervention

The UK Medical Research Council (MRC) framework for the development and evaluation of complex interventions for randomized control trials (RCT) is used as a theoretical guide to designing the intervention [6]. The MRC framework allows for the development of a high-quality study design, execution, generalizability of the results, and outlines five key phases for intervention development: a preclinical/theoretical phase, a modeling phase, a phase of exploratory trials prior to the randomized controlled trial (RCT), the trial itself, and long-term implementation [36]. All phases except phase two (exploratory trial phase) and phase five (long-term implementation phase) are incorporated here. A detailed overview is provided in Table 1.

**Table 1:** The MRC Framework applied for the development and evaluation of a complex intervention in diabetes care.

**Phases**			
**Phase I- Preclinical theory (Why should the intervention work?)**			
**Content**	**Methods**	**Results**	**Publications**
- Collecting evidence on the effectiveness of multifaceted diabetes intervention programs – Identification of evidence on appropriate outcome indicators - Influence of local context	Review of systematic reviews on diabetes care programs in primary care, outpatient, community and hospital settings to identify: conceptual backgrounds of programs, goals, settings, type of program, type of interventions, type of indicators, (cost) effectiveness of programs and interventions	Overview of best choice of interventions and indicators, selection of conceptual model, overview of major confounders, overview of strategic design issues, overview of barriers to high-quality diabetes care at the macro, meso and micro level	[37]
			
**Phase II- Modeling (How does the intervention work?)**			
**Content**	**Methods**	**Results**	**Publications**

- Understanding of the pathways by which the problem is caused and sustained - Exploration of whether the pathways are amenable to change and, if so, at which points - Quantification of the potential for improvement (estimates of likely effect size) - Program development	- Stakeholder interviews to identify and understand barriers to high-quality diabetes care in the Belgian health care system and multidisciplinary team meetings to discuss program development	- Definition a multifaceted intervention/implementation strategy and outcome-indicators and local adaptation of the treatment protocol	[41]
			
**Phase III – Exploratory Trials (not performed)**			
			
**Phase IV – Randomized Controlled Trial**			
The Diabetes Project Leuven (cluster randomized trial)			
			
**Phase V – Long Term Implementation (not performed)**			

### Preclinical phase of the MRC framework

This phase involves exploration of the relevant theory and evidence to refine the underlying hypotheses, conceptual model, interventions, and indicators. We have previously performed a review of systematic reviews for this purpose [37]. A total of 21 systematic reviews (1989–2006) were included in the review and represented 185 diabetes care programs. Conceptual background, goals, settings, type of programs, type and number of interventions, type and number of indicators, and (cost) effectiveness were evaluated in both the 21 systematic reviews and the individual diabetes care programs. The program is further built on the CCM [8,9] and principles of integrated care. As there is no unambiguous definition of integrated care, we further build on the definitions of Ellrodt and colleagues [38], Mur-Veeman and colleagues [39], and the Disease Management Association of America (DMAA) [40]. We consider integrated care as 'an organizational process of continuous coordination of evidence-based and relevant interventions across the entire health care delivery system and care continuum that seeks to maximize quality of care tailored to the needs of every individual patient while minimizing costs'.

Besides exploring relevant theory and evidence, the local context in terms of existing national and regional governmental policies, characteristics of the region, and perceived barriers to high-quality diabetes care were extensively studied with regard to their impact on the content and execution of the study protocol. We have previously organized stakeholder interviews, including a representative group of 18 Belgian opinion leaders and experts in diabetes care [41].

### Modeling phase

In the modeling phase, we delineated the components of our complex intervention and the underlying mechanisms by which they influence the outcomes. We sought to understand the pathways by which the problem is caused and sustained, including all barriers to high-quality diabetes care. We also explored whether the pathways were amenable to change, and if so, at which points. Finally, we estimated potential for improvement in both process and primary outcomes. This analysis produced the best achievable combination of intervention components, implementation strategies, and intensities of care delivery, as well as the identification of feasible and valid outcome measures.

### Interventions

Two separate groups are defined: the first group will receive a usual quality improvement program (UQIP), and a second group will receive an advanced quality improvement program (AQIP). Physicians can make use of program services on a voluntary basis.

The UQIP arm will aim to improve adherence to evidence-based guidelines and to reduce the rate of clinical inertia in PCPs. The term 'usual' is applied because these interventions address the principal factors contributing to clinical inertia (physician, patient, and office system factors) and represent standard requirements for what is considered quality of diabetes care in most health care systems according to international clinical guidelines [42], and theoretical frameworks on quality of diabetes care in particular [43]. The first intervention arm is innovative to the Belgian healthcare system and adds to available insights from the international literature on how to address clinical inertia in diabetes care.

The AQIP arm will receive similar interventions, but will also include supplementary and experimental interventions that extensively focus on behavior changes in patients and providers. Interventions that focus on the patient aim at a more active involvement of the patient in his/her treatment regimen, with a special focus on lifestyle attitude changes. Improvements in 'patient empowerment' will further decrease clinical inertia by increasing the patient's willingness to intensify his/her treatment [44,45]. Interventions that target the PCP focus on improvements in communication patterns with patients, interdisciplinary shared care, and involving PCPs in community campaigns. This multi-factorial approach, with a focus on patient, provider, and organizational aspects of care, is fully in line with the latest insights and findings on high-quality chronic care, and high-quality diabetes care in particular [9,19,46-53].

The differences between the AQIP and the UQIP are outlined further below and in Table 2.

**Table 2:** Overview of components of the Usual Quality Improvement Program (UQIP) and Advanced Quality Improvement Program (AQIP).

**Patient**		
Lack of adhere to treatment regimen and clinical inertia related to:*e.g*. Limited motivation or resistance to adopting lifestyles that support optimal disease care.		
	Usual Quality Improvement Program (UQIP)	Advanced Quality Improvement Program (AQIP)
Patient education	Medical assessments and education upon referral of the PCPs by diabetologist or DCT	Medical assessments and education upon referral of the PCPs by diabetologist or DCT (DCT)
	= internist, nurse educator, dietician and ophthalmologist	= internist, nurse educator, flying educator, dietician, ophthalmologist and health psychologist
Promotion of self-management	----	Education of patients in practice (by flying educator)
	----	Education at patient's home (by flying educator)
	----	Counseling by health psychologist
	----	Structured educational materials from DCT
	----	Structured educational materials from community organizations
	----	Group educational sessions for patients and family members
	----	Free access to blood monitoring tools for self-management

**Professional**		
**Lack of adherence to guidelines and clinical inertia related to:*e.g*. Overestimation of care actually delivered, a failure to identify and manage comorbid conditions, unawareness or disagreement with evidence-based goals of care and 'soft reasons' to avoid intensification of therapy**.		
	Usual Quality Improvement Program (UQIP)	Advanced Quality Improvement Program (AQIP)
Clinician education	Distribution of treatment protocol	Distribution of treatment protocol
	Two post-graduate educational sessions	Four post-graduate educational sessions provided by diabetologist (opinion leader):
	Evidence based guidelines	Evidence-based guidelines and principles of shared care
	The use of insulin	The use of insulin
		Patient-centered counseling
		Peer review
	Standard educational materials	Extended educational materials
	----	Inviting PCPs during DCT meetings to discuss patient cases
	----	Providing structured communication forms to PCPs by DCT
	----	Distribution of shared care protocol + referral indication
Feedback	At start and end of project: summary of clinical performance	Every 3 months: summaries of clinical performance
	----	Every three months: benchmarking feedback
Reminders	Clinical reminders at start and end of project	Every three months: Clinical reminders
	----	Every three months: Shared care reminders
**Organisational**		
Lack of office system support and organizational aspects of care related to clinical inertia:*e.g*. Lack of decision support and a team approach to care.		
	Usual Quality Improvement Program (UQIP)	Advanced Quality Improvement Program (AQIP)
Team changes	DCT operating close to regular care	Active instalment of DCT operating under supervision of a diabetologist from a University Hospital
		Diabetes Program manager providing logistic support to PCPs
	----	Introduction of shared care protocol Active encouragement by DCT and scientific team of PCPs to use shared care protocol
	----	Referral arrangements Active encouragement by DCT and scientific team to adhere to referral arrangements
	----	Liaison activities by DCT towards in-hospital DCT in secondary care
	----	Involvement of independent pharmacists
Continuous quality improvement	Quality Assurance Team	Quality Assurance Team

We will use two classification schemes to incorporate all six dimensions of the CCM based on the classification scheme from Shojania and colleagues [54], who defined eleven distinct categories of quality improvement interventions adapted from the Cochrane Effective Practice and Organization Of Care (EPOC) group [55]. These categories are: patient education, promotion of self-management, clinician education, audit and feedback, case management, team changes, electronic patient registry, clinician reminders, facilitated relay of clinical information to clinicians, patient reminder systems, and continuous quality improvement. Five interventions are not included in the service program as they are either integrated in other interventions of the program (*e.g*., the patient reminder system is integrated with physician reminder system) or because of complexity in the Belgian primary health care system (case management, audit, electronic patient registry, and facilitated relay of clinical information to clinicians). The different implementation strategies are derived from an overview by Grol and Wensing [56], who have summarized thirteen important theories and models related to the implementation of change to improve diabetes care. These theories/models relate to individual professionals/patients, the social context, and the organizational and economic context.

### Level one: patient

#### Patient education/promotion of self-management

Both patients in the AQIP and UQIP arms can be referred by their PCP to a DCT to receive a medical assessment by an internist as well as to receive patient education, dietary advice, and examination by an ophthalmologist. This core membership of internists, nurse educators, dieticians, and ophthalmologists reflects the basic requirements of diabetes treatment: nutrition, medication, self-monitoring, self-management, and the management of risk factors [57]. Physicians from both the AQIP and UQIP can ask internists/diabetologists for advice on complex patient cases, with or without patient referral. Educational services and promotion of self-management to patients of the AQIP and UQIP are only provided upon referral of the physician. Nurse educators have received a post-graduate one-year training program on diabetes nursing care. The nurse educator applies individual patient counseling, didactic goal-setting, and situational problem-solving as key educational methods to patients in the AQIP, whereas patients from the UQIP receive services approximating regular care, *i.e*., individual patient counseling. Physicians from both the AQIP and UQIP can consult dieticians for complementary dietary advice or can refer their patients to discuss information on meal algorithms, dietary strategies, and tailoring food intake to meet the patients' lifestyle, motivation, and specific needs [57]. Education on lifestyle changes, identification of barriers to diabetes self-management, and stress management will be provided by a health psychologist to patients in the AQIP-program after physician referral.

Patients in the AQIP can receive additional services, including group educational sessions for both patients and relatives, education at home or at the physician's practice (provided by a traveling educator), structured and printed educational materials from the DCT and community organizations, and free tools for self-monitoring of blood glucose levels.

### Level two: professional

#### Clinician education

Interventions for clinician education include an increased understanding of principles guiding clinical care or awareness of specific recommendations for the patient population using a treatment and shared care protocol, as well as four post-graduate educational sessions based on the Transtheoretical Model of Change [58]. The first session will involve training on the use of evidence-based guidelines and the principles of shared care. A second and a third session will focus on the use of insulin and patient-centered counseling. A fourth session will be set up as a peer review session. Educational messages are delivered, for most part, by a locally well-known diabetologist ('opinion leader') using techniques of group academic detailing [59]. Providing clinical leadership in secondary care is important for PCPs working in an unstructured and thus non-integrated health care environment.

The UQIP will incorporate only the first two sessions. AQIP physicians can attend all four sessions and will also receive extended educational materials. Physicians from both groups will receive accreditation points from a national system for their participation at the educational sessions.

### Feedback

Feedback interventions, provided by a program manager to the physicians, will include summaries of clinical performance of diabetes care delivered to individual patients over a three-month period. AQIP physicians will receive ongoing benchmarking feedback, whereas the UQIP will only receive benchmarking feedback at the start and end of the project. Feedback includes the percentage of a physician's patients who achieve target levels for glycosylated hemoglobin, LDL, total cholesterol and triglycerides, systolic/diastolic blood pressure, an eye and foot examination, aspirin and statin prescriptions, anti-hypertensive medication, smoking status, and weight loss.

### Clinician reminders

Clinician reminders for physicians of the AQIP are combined with quarterly feedback by the program manager and reminders to make use of the DCT if treatment targets are not met. Physicians are asked to remind their patients about upcoming appointments. Patients are asked by the physicians to make use of a diabetes passport in which the appointments are noted together with important treatment results. Physicians of the UQIP do not receive clinician/patient reminders nor do they receive reminders on the use of the DCT.

### Level three: organisational

#### Team changes

Team changes are operationalized in three ways. Initially, a DCT will be installed in two primary care facilities that are run by PCPs. The DCTs will be intensively supervised by a diabetologist from the academic hospital in the project region who provides clinical leadership to the team. All DCT members will receive a 60-hour in-house training program on the use of a shared care protocol, communication skills, and team dynamics. Key elements of the interdisciplinary team include shared leadership with common goals, shared professional identity, and collaborative, rather than consultative, relationships among members [60]. Team members are expected to engage and learn from each other and to attend scheduled meetings. An experienced counselor and a member of the academic project team will oversee the training program. The DCTs operate in support of the PCPs and actively promote referrals to physicians of the AQIP if treatment targets are not met [61]. Fortnightly interdisciplinary meetings will be organized between the members of the DCT who can invite individual physicians from the AQIP to discuss complex patient conditions.

Nurse educators, dieticians, and the health psychologist will meet their colleagues from a university hospital-based diabetes team and the supervising diabetologist on a quarterly basis to exchange experiences and discuss complex patient cases. Internists will meet with the supervising diabetologist every other month to discuss individual patient cases.

Structured, extensive reports will be provided by members of the DCT to the AQIP because PCPs rank standardized, structured correspondence very high [62,63]. Physicians of the UQIP will only receive standard communication forms.

Team changes will also include the active promotion of a diabetes program manager who operates as the central point of referral for the physicians. The program manager will be selected based on the following criteria: strong interpersonal communication skills, the ability to create trust, knowledge of diabetes, and organizational capabilities. The program manager will provide physicians from the AQIP with extended (logistic) support, including physician reminders, providing feedback, liaison activities between the DCT and physicians, organizing group educational sessions, and responding to questions on the study or diabetes-related topics. A project website to facilitate this will be accessible for AQIP and UQIP physicians.

The final team change will be involvement of independent pharmacists in the study. Pharmacists are asked to provide physicians in the AQIP program with medication schemes of their patients upon request. As such, pharmacists can play a more active role in patient monitoring or adjusting medication regimens [64-66].

### Continuous quality improvement

Continuous quality improvement will be assured by an iterative process for assessing quality problems in the implementation of the project, developing solutions to those problems, testing their impacts, and then reassessing the need for further action. For this purpose an interdisciplinary quality assurance team will be established that includes a diabetologist, four PCPs, two nurses, internists, dieticians, and pharmacists. The quality assurance team will be asked to monitor the implementation of the project, as well as evaluate outcome indicators of the project. Meetings will be organized on a regular basis with individual members of the quality assurance team.

### Sample size

The project funding agency requires a sample size of at least one-third of the potential PCPs (n = 379), which would capture roughly 2,500 patients with type 2 diabetes mellitus. This sample size allows 80% power (type II error: 0.20) to detect a 20% relative difference between the intervention arms in the proportion of patients achieving a 10% improvement in any one of the following: blood pressure, total cholesterol, or HbA1c (type I error: 0.05; assumed intracluster coefficient 0.6; [67] for calculation methods).

### Randomization and allocation concealment

After recruitment, a researcher not involved in the study and blind to the identity of the practices will perform a randomization (by computer-generated numbers) stratified by practice size (solo/duo/group practice) and the presence or absence of an electronic medical recording system. To minimize the possibility of selection bias, all patients within a cluster will be included. Blinding will be ensured for the participating patients, but is not possible at the physician level.

### Data collection

These practices have no pre-existing registers of diabetic patients. Patients with type 2 diabetes mellitus will be identified using physician memory, searching computerized records, and laboratory lists of patients with increased glycemia or registered glycosylated hemoglobin. Baseline data will be collected over a seven-month period. PCPs will be asked to perform a complete examination and blood analysis at the patient's first visit and to complete a paper form. Identified patients without a visit during the first three months of the project will be invited to participate. The completeness of data capture will be double-checked by a data monitor. Final data will be collected over a seven-month period, with call-backs for non-compliant patients. Patient data sheets include socio-demographic and biomedical data. PCPs will need to indicate whether diabetes is treated by the PCP or in a diabetes clinic.

### Primary and secondary endpoints

The primary endpoints of the study are the proportion of patients reaching ADA targets for three clinical outcomes: HbA1c < 7%; SBD ≤ 130 mmHg; and LDL-C < 100 mg/dl. Secondary endpoints are the mean improvements in individual values of 12 validated parameters: HbA1c, LDL-C, HDL-C, Total Cholesterol, SBP, DBP, weight, physical exercise, healthy diet, smoking status, and statin and anti-platelet therapy.

### Statistical analysis

Statistical analyses will be performed using an intent-to-treat approach with a multilevel model. Linear and generalized linear mixed models will be used to account for the clustered nature of the data, *i.e*., patients clustered within PCPs, and repeated assessments clustered within patients. Such models measure how outcomes change over time within patients and whether these changes depend on patient and/or PCP's characteristics, such as the intervention program or DCT use (see hypothesis two). DCT use is defined as having at least one consultation with a member of the team besides the health psychologist and the traveling educator, which are only available for AQIP patients.

We will use generalized estimating equations (GEE), an extension of the quasi-likelihood approach, to test for differences in the evolution of the primary and secondary endpoints for all patients and within the intervention arms. For binary variables, we use the exponential inverse transformation to obtain the 95% confidence interval for the odds ratio.

Subgroup analyses (see hypothesis one) can distinguish intervention effects using different cut-off values. For HbA1C, three subgroups are defined: patients with HbA1c < 7%; HbA1c ≥ 7% and < 8%; and HbA1c ≥ 8%. For SBP, four subgroups are defined: patients with SBP ≤ 130 mmHg; SBP > 130 mmHg and ≤ 140 mmHg; SBP > 140 mmHg and ≤ 160 mmHg; and SBP > 160 mmHg. For LDL-C, four subgroups are defined: patients with LDL-C < 100 mg/dl; LDL-C ≥ 100 mg/dl and < 115 mg/dl; LDL-C ≥ 115 mg/dl and < 130 mg/dl; and LDL-C > 130 mg/dl.

Linear mixed models with subject-specific intercepts and slopes are used to test whether subject-specific evolutions are related to initial parameters. HbA1c will be transformed logarithmically to meet the parametric assumptions of the statistical models. All analyses will be performed using SAS, version 9.

## Discussion

Trials of complex interventions inform the drive to provide the most cost-effective health care [7]. RCTs are recognized as the 'gold standard' methodology in quantitative research. Health care interventions are, however, often complex and are always implemented in complex health care settings [68-71]. Complex interventions often have particular characteristics that reduce chances of success in a RCT, including the incorporation of multiple components, targeting multiple outcomes, being difficult to implement or evaluate, or aiming to achieve outcomes that are notoriously difficult to influence [72]. In this context, the complexity of an intervention can present a substantial barrier to its adoption [73]. Complex interventions therefore have greater scope for variation in their delivery and are more vulnerable to one or more components not being implemented correctly [74].

Although we have not performed a pilot trial to assist in data interpretation or clarify process and outcome results, our stakeholder analysis informed our understanding of existing barriers to high-quality diabetes care and allowed us to incorporate innovative change interventions, such as interdisciplinary teams operating on the primary/specialty care interface and educational strategies that target changes in professional practice and improvements in patient empowerment [75]. These hypotheses will be tested using a large group of physicians and patients over an 18-month period. Most quality improvement programs include smaller target groups and shorter intervention periods of six months, which may not be long enough to completely remove the Hawthorne effect. Our study also targets the primary/specialty care interface, an important attribute of high-quality diabetes care [76]. In particular, the clinical leadership and coaching provided by a diabetologist to both the PCPs and the DCT is of particular importance in fragmented systems of care, such as in Belgium. We also explicitly focus on multiple cardiovascular risk factors as the primary outcomes, whereas other studies have not [77]. Finally, we incorporate all six dimensions of the CCM,, and are only the fourth study in diabetes care to do so [9,78,79]. The use of all six dimensions of the CCM permits evaluation of how CCM components are associated with improved outcomes to further refine the model. We therefore explicitly describe how the implementation strategies relate to every dimension of the CCM. Implementation strategies in complex interventions are rarely described [80], even in large-scale implementation studies, which limits the understanding of why an intervention is or is not locally successful [81].

## Competing interests

The authors declare that they have no competing interests.

## Authors' contributions

BL, GG, and VDBC participated in the study design and drafted the manuscript. MC, AB, VG, CA, IA, GR, and HJ participated in the study design. All authors have read and approved the final manuscript.
